# Plasticity of fruit and oil traits in olive among different environments

**DOI:** 10.1038/s41598-019-53169-3

**Published:** 2019-11-18

**Authors:** Soraya Mousavi, Raul de la Rosa, Abdelmajid Moukhli, Milad El Riachy, Roberto Mariotti, Mariela Torres, Pierluigi Pierantozzi, Vitale Stanzione, Valerio Mastio, Hayat Zaher, Abderraouf El Antari, Salam Ayoub, Faten Dandachi, Hiyam Youssef, Nikolas Aggelou, Cibeles Contreras, Damián Maestri, Angjelina Belaj, Marina Bufacchi, Luciana Baldoni, Lorenzo Leon

**Affiliations:** 1 CNR - Institute for Agricultural and Forest Systems of the Mediterranean, 06128 Perugia, Italy; 2 CNR - Institute of Biosciences and Bioresources, 06128 Perugia, Italy; 3 IFAPA - Centro Alameda del Obispo, Córdoba, Spain; 4 INRA - CRRA, Marrakech-Safi, BP 533, Marrakech, Morocco; 5 LARI - Lebanese Agricultural Research Institute Tal Amara, Bekaa, Lebanon; 6 Estación Experimental Agropecuaria San Juan (EEA INTA San Juan), and CONICET. Ing. Marcos Zalazar (Calle 11) y Vidart. Villa Aberastain, Pocito, 5427 San Juan Argentina; 7 National Agricultural Research Center (NARC), Baqa, 19381 Jordan; 8 MAICh - Department of Horticultural Genetics and Biotechnology, 73100 Chania-Crete, Greece; 9 Instituto Multidisciplinario de Biología Vegetal (IMBIV, CONICET-UNC). Av. Vélez Sarsfield 1611, X5016GCA, Córdoba, Argentina

**Keywords:** Natural variation in plants, Plant breeding

## Abstract

Olive is a long-living perennial species with a wide geographical distribution, showing a large genetic and phenotypic variation in its growing area. There is an urgent need to uncover how olive phenotypic traits and plasticity can change regardless of the genetic background. A two-year study was conducted, based on the analysis of fruit and oil traits of 113 cultivars from five germplasm collections established in Mediterranean Basin countries and Argentina. Fruit and oil traits plasticity, broad‐sense heritability and genotype by environment interaction were estimated. From variance and heritability analyses, it was shown that fruit fresh weight was mainly under genetic control, whereas oleic/(palmitic + linoleic) acids ratio was regulated by the environment and genotype by environment interaction had the major effect on oil content. Among the studied cultivars, different level of stability was observed, which allowed ranking the cultivars based on their plasticity for oil traits. High thermal amplitude, the difference of low and high year values of temperature, negatively affected the oil content and the oleic acid percentage. Information derived from this work will help to direct the selection of cultivars with the highest global fitness averaged over the environments rather than the highest fitness in each environment separately.

## Introduction

Cultivated olive (*Olea europaea* L. subsp. *europaea* var. *europaea*) covering more than eight million hectares, is one of the main oil crops worldwide. The crop, traditionally cultivated in the Mediterranean Basin, where it still represents 92% of the entire world supply (International Olive Council data), is now experiencing a great expansion to new and different areas of south-western Asia, Oceania, South Africa, and the Americas^1–3^. The Mediterranean area, considered particularly sensitive to global climate change, represents a hotspot region where the effects of new climate conditions will threaten the agro-ecosystems, with serious impacts on plant/environment interactions^4–6^. Thus, olive cultivation is going to face the challenges posed by the new climate constraints, that will benefit some olive-producing areas, and will adversely affect others^6,7^. In this context, knowledge about the olive reaction to the new climate scenarios represents relevant information for defining new adaptation strategies^8^.

The ability of plants to face environmental changes, expressing different phenotypes under various climate constraints, is known as phenotypic plasticity^9,10^. The term is used to describe the physiological, morphological and developmental variability of a given genotype in response to different environments^11,12^. The extent of genotype plasticity may increase or reduce plant adaptation towards specific conditions^13–19^. The genotypic variation, within each crop species, could contribute to overcome local constraints through the selection of individuals suitable for each condition. In fact, plant crop species show different responses across different climates^20^. The new olive plantation areas, outside the Mediterranean countries, require greater knowledge on the performance of this crop under different climatic conditions^21,22^. Global warming can affect olive yield and oil quality, therefore the study of oil composition comparing new and traditional cultivation areas can provide useful information to optimize the crop productivity^23^.

Olive trees are able to survive and produce under different and complex agro-ecological conditions, because of the high genetic variability within the species^20,23–29^. Therefore, it may work as a reference plant species among several fruit perennial crops. Olive cultivation could contribute mitigating the effects of climate change as: water scarcity, increasing temperatures and meteorological disorders^30,31^. While the increase of mean temperatures in recent years has made possible to extend the olive cultivation to northern latitudes, the trend of further increases in temperatures could cause the progression of pre-desertification conditions in Mediterranean countries, making its cultivation more arduous^6,32,33^. On the contrary, sudden thermal drops, that could occur in areas highly suited to olive growing, could lead to the lower crop yields^3,34^. In fact, the olive cultivation area has changed over the millennia, depending on several factors, among which the climate conditions played a main role^35,36^.

In olive, fruit oil content and composition is the result of a complex interaction between genotypic, environmental and agronomical factors^34–36^. Genotype by environment (G × E) interaction results by variable phenotypes, but informative data on the correlation between genotype and phenotype and on the effectiveness of empirical selection are still missing^1,37–40^. G × E interaction also makes it difficult to choose cultivars well adapted to new and untested climate conditions^9^. Fruit size, oil content and oil quality are target objectives of the olive breeding^41–44^. Deep knowledge of the possible environmental effects on these traits would be useful to allow for an effective selection of high performing genotypes. Regarding oil quality, the unsaturated fatty acids (FAs) are the most important component of olive oil, followed by secondary metabolites, such as phenols^45,46^. Olive oil accumulates in the drupe mesocarp (more than 95%) and just a minimal percentage in the seed^47^. This feature determines a greater susceptibility of fatty acid composition to thermal variations concerning seed oleaginous crops^48^. Olive oil contains up to 83% of the monounsaturated oleic acid, followed by linoleic (3.5–21%), palmitic (7.5–20%), stearic (0.5–5%) and linolenic acid (<1%). The low content of saturated FAs in olive oil and the high percentage of monounsaturated FAs makes it an excellent source of fat, able to reduce cardiovascular diseases^49^.

Among the climatic factors that affect FAs composition, temperature plays an essential role by regulating FA desaturases^50–52^ and, therefore, variations in temperature may cause modifications in FAs composition^53^.

Several studies have evaluated the effect of climate conditions on fruit maturation and oil composition in restricted sets of cultivars and locations^53–55^, nevertheless, it has been observed that during oil accumulation, olive varieties could have different responses to temperature in terms of FAs composition^38,51,52,54,56^. Moreover, other experiments, under controlled temperature conditions, demonstrated that oil content is sensitive to temperature changes. In fact, by increasing the daily mean temperature between 16 °C and 32 °C, oil content decreases^23^. Analyzing oil accumulation on six olive cultivars at different locations, a negative correlation was observed between oil content and mean temperature^37^ and between maximum daily temperature during oil accumulation within a narrow range (29–31.5 °C) of temperatures^18,57^.

A comprehensive study testing a large number of cultivars in different locations with diverse climate conditions has never been previously reported. This work represents the first extensive study on the evaluation of a very large set of olive cultivars (113) during two growing seasons from five locations geographically and climatically different in Italy, Spain, Morocco, Lebanon, and Argentina. These cultivars were assessed for FAs composition, oil content and fruit traits. Results should address the following questions: (i) how do cultivars respond to different environments? (ii) How different environments may affect fruit and oil traits in olive? (iii) How knowledge on cultivar plasticity for FAs composition may be applied to optimize orchard management and breeding strategies to improve olive adaptation under new climate constrains?

## Results

### The environmental effect on trait plasticity in a common set of cultivars

**Figure 1:**
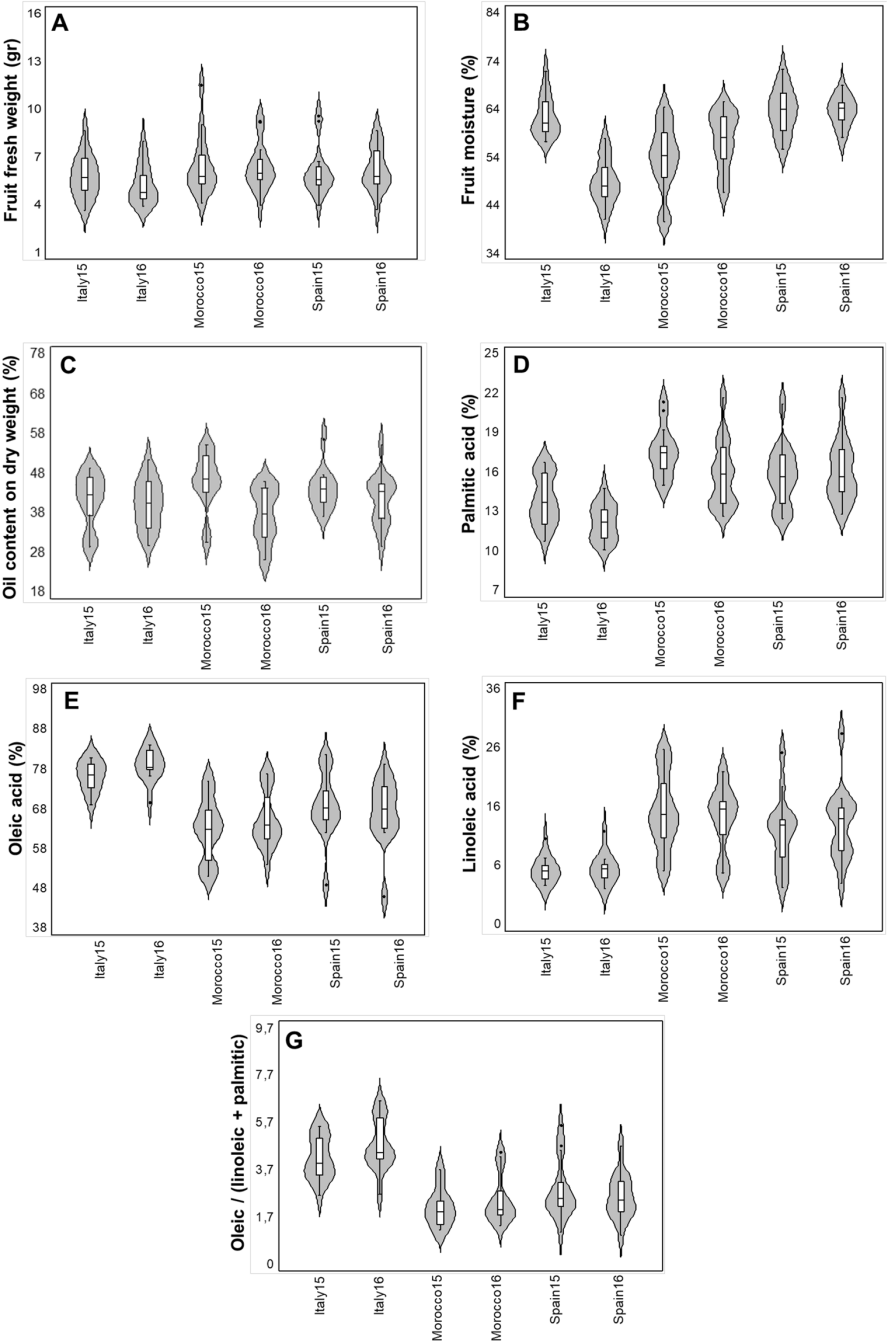
Violin graphs representing trait variation in each environment (three sites x two seasons). Each plot shows the distribution of data for fourteen cultivars from the minimum to the maximum level, with horizontal inner lines showing the data median. The white boxplots representing the lower and upper limits of the first and third quartiles. The outliers are indicated with black dots. The horizontal width of the violin depends on the data density.

A wide range of variation was observed for all analyzed traits. The traits included: fruit fresh weight (FrFW), fruit moisture (FrM), oil content in fruit dry weight (OCFrDW), palmitic acid (C16:0, expressed in percentage), oleic acid (C18:1), linoleic acid (C18:2) and oleic/(linoleic + palmitic) acids ratio (OLP) for the common set of 14 cultivars evaluated in six environments (Fig. 1, Supplementary Table S1). Among the studied characters, FrFW, C18:2, and OLP were the most variables within each environment, while FrM and C18:1 showed the lowest variability (Supplementary Table S1).

The highest average value of FrFW trait was related to Morocco15 (Morocco 2015) and Morocco16 (3.4 and 3.3 g, respectively), while in Morocco15 was detected the highest variability of this trait (55%) (Fig. 1A, Supplementary Table S1).

The highest average values of FrM were found in Spain15 (63.5%), whereas its main variation was related to Morocco15 (Fig. 1B and Supplementary Table S1).

The highest average values of OCFrDW were related to Morocco15 (45.8%), while the highest variation was related to Morocco16 (Fig. 1C, Supplementary Table S1).

The highest average values of C16:0 were registered for Morocco15 and Spain16 (17.5 and 16.1%, respectively), although the highest variation was registered in Morocco16 (Fig. 1D, Supplementary Table S1).

For C18:1, the highest average value was observed in Italy16 (79.1%), while the highest variation was found for the Morocco15 (12.2%) (Fig. 1E, Supplementary Table S1). A general lower variability for FAs composition was observed in Italy15 and Italy16, related to IOGC in Italy respect to the rest of locations, particularly for C18:1.

The highest average values of C18:2 were found for Morocco15 (15.2%), whereas its highest variation was related to Spain15 (Fig. 1F, Supplementary Table S1).

Finally, the OLP ratio had the highest values in Italy15 and Italy16, while approximately the same ratios were observed in the other four environments. The highest variation of OLP among different environments was related to Spain15 with 42.6% (Fig. 1G, Supplementary Table S1).

### The genotype effect on trait plasticity in a common set of cultivars

**Figure 2:**
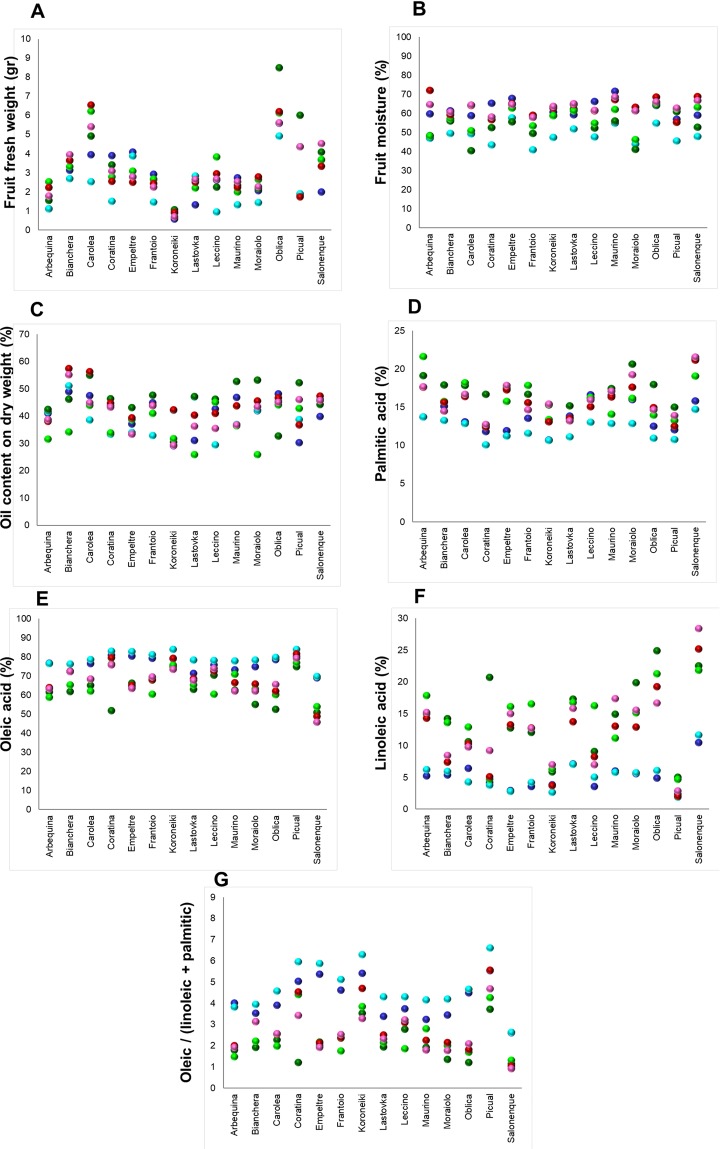
Scatter plots showing trait variation for each cultivar over six environments (blue - Italy15, light blue - Italy16; green - Morocco15, light green - Morocco16; red - Spain15 and pink - Spain16). Each colored circle is the mean trait value of two trees for each cultivar. The x axis in all graphs represent the independent variable (cultivar), the y axis of the graph corresponds to the trait value.

The same dataset of 14 cultivars in six environments was used to investigate the cultivar variability (Fig. 2 and Supplementary Table S2). In this sense, the highest average value of FrFW was registered for cv. ‘Oblica’ (Fig. 2A and Supplementary Table S2). The highest variation for FrFW, in six different environments, was registered for cvs. ‘Picual’ and ‘Leccino’ (48.6 and 33.8%, respectively) whereas cv. ‘Bianchera’ was the most stable for this trait.

The highest average values (%) of FrM were related to ‘Oblica’ and ‘Maurino’ cultivars (63.9 and 63.4%, respectively), while ‘Frantoio’ and ‘Moraiolo’ cultivars displayed the lowest ones. Among the 14 cultivars, ‘Arbequina’ and ‘Moraiolo’ were the most unsteady cultivars for this character (CV_trait_, 16.8 and 17.4%, respectively) (Fig. 2B and Supplementary Table S2).

The highest average percentages of OCFrDW were related to cvs. ‘Bianchera’ (49.6%) and ‘Carolea’ (47.8%), while ‘Koroneiki’ showed the lowest values among all cultivars. In the six environments under study, ‘Lastovka’ and ‘Moraiolo’ cultivars displayed the highest variation for this trait (Fig. 2C and Supplementary Table S2).

The highest amount of C16:0 was detected for ‘Salonenque’ cultivar (19.0%), while ‘Coratina’ was the cultivar which had the lowest amount (12.7%) (Fig. 2D and Supplementary Table S2). C16:0 had the highest variation in the cvs. ‘Empeltre’ (17.6%) and ‘Arbequina’ (16.4%).

‘Picual’ and ‘Koroneiki’ cultivars showed the highest amount of C18:1, with 79.4 and 77.8% on average, respectively. It is interesting to note that ‘Picual’ not only reached the highest amount of C18:1, but also was the most stable cultivar for this trait in all environments (3.7%) (Fig. 2E and Supplementary Table S2). While ‘Salonenque’ and ‘Oblica’ cultivars showed the highest variation for this trait (16.8% and 14.7%, respectively).

The cultivar ‘Salonenque’ had the highest average amount of C18:2 (20.0%), while the lowest values were detected in ‘Picual’ (3.2%). ‘Coratina’ cultivar showed the highest variation for C18:2 (75.4%), while ‘Carolea’ was the most stable cultivar for this trait (31.6%) (Fig. 2F and Supplementary Table S2).

The highest OLP ratio was found in csv. ‘Picual’ (5.1) and ‘Coratina’ (4.1), while the lowest amount was observed in ‘Salonenque’ cultivar. ‘Oblica’ and ‘Empeltre’ cultivars had the highest percentage of instability for OLP (51.7 and 51.9%, respectively), being ‘Picual’ the most stable one for this trait (Fig. 2G and Supplementary Table S2).

Finally, the most variable traits over six environments were C18:2 and OLP which showed the highest CV_m_ values (40.75% and 32.65%, respectively). In contrast, OCFrFW (8.41%) and C18:1 (9.04%) were the most stable traits over six environments (Supplementary Table S2).

Pearson correlation coefficients among the evaluated traits by cultivar showed significant negative correlation coefficients between C18:1 *versus* C16:0 and C18:2 (Supplementary Table S3). These results were highly consistent among the different evaluated cultivars.

### The complex effect of genotype, environment and their interaction on trait plasticity of all studied cultivars

A full dataset of all cultivars (113) from nine environments (Supplementary Table S4) was used to analyze the variance components using REML models (Table 1). σ²_G_ was the main provider to total variance for FrFW (64.6%) and C16:0 (47.3%), σ²_E_ for FrM (69.9%) and OLP (43.9%), and σ²_GxE_ for OCFrDW (43.6%). The remaining FAs evaluated (C18:1 and C18: 2), both σ²_G_ and σ²_E_ showed similarly high values (from 35.8% to 39.1%). Consequently, broad-sense heritability (H^2^) ranged from 0.32 to 0.73, over the seven studied traits. FrFW had the highest H^2^ estimates (0.73); other traits, especially FAs and OLP, had also high values (0.59–0.64), while the lowest one was related to OCFrDW (0.32).

**Table 1:** Results of variance analysis (%) and heritability from REML models for 113 cultivars in nine environments.

	FrFW	FrM	OCFrDW	C16:0	C18:1	C18:2	OLP
Genotype (σ²_G_)	64.6	13.6	27.2	47.3	36.1	39.1	35.9
Environment (σ²_E_)	11.5	69.9	16.6	26.1	38.6	35.8	43.9
Genotype x Environment (σ²_GxE_)	18.4	10.8	43.6	11.1	14.9	15.5	12.4
Residual Variance (σ² _ε_)	5.5	5.7	14.6	15.6	10.4	9.6	7.7
H^2^	0.73	0.45	0.32	0.64	0.59	0.61	0.64

Abbreviations: FrFW, fruit fresh weight, FrM, fruit moisture, OCFrDW, oil content in fruit dry weight, C16:0, palmitic acid, C18:1, oleic acid, C18:2, linoleic acid and OLP, oleic/(linoleic + palmitic) acids ratio.

H2 = broad sense of heritability.

**Figure 3:**
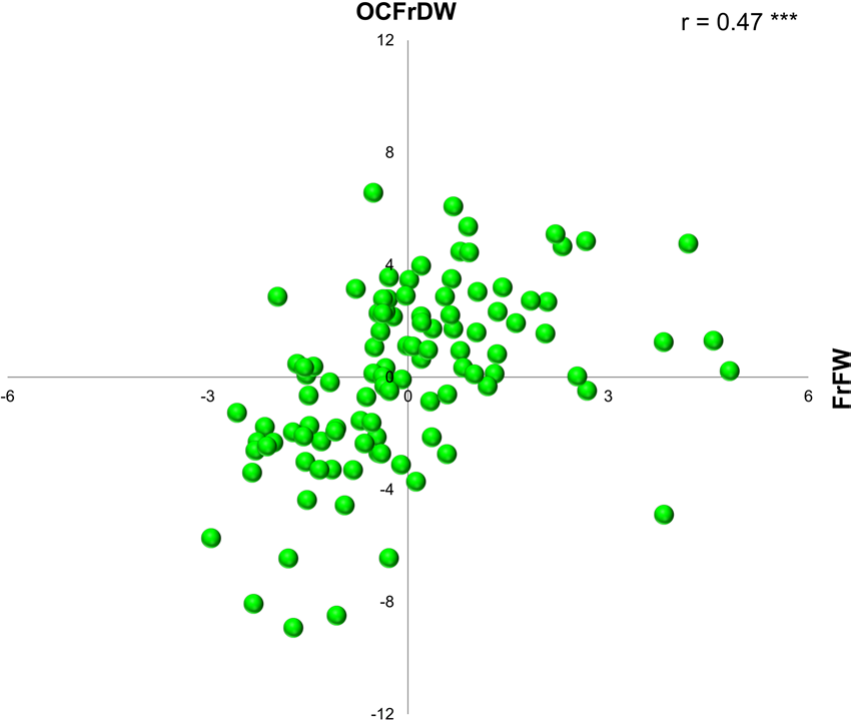
The best linear unbiased prediction (BLUP) graph for the effect of genotype on OCFrDW (oil content in fruit dry weight) *vs* FrFW (fruit fresh weight) interactions.

BLUP values obtained for three of the main evaluated traits (OCFrDW, FrFW, and OLP), representative of the overall observed variability, were used to study the relationships among them and to identify promising genotypes regarding these traits. A positive significant correlation was obtained between genotypic BLUP values for OCFrDW and FrFW. Some of the analyzed cultivars, such as ‘Konservolia’, ‘Chalkidikis’ and ‘Domat’ had higher OCFrDW and FrFW than the average, while others such as ‘Koroneiki’, ‘Megaritiki’, ‘Pendolino’ and ‘Vera’ showed high negative values (Fig. 3). Correlation between BLUP values for OCFrDW and OLP was not significant, and then the distribution of cultivars for these traits was approximately the same in all BLUP areas (Supplementary Fig. S1).

BLUP values for OCFrDW, FrFW and OLP traits on the different environments were not correlated among them, indicating different best-performing environments for each of the evaluated traits (Supplementary Fig. S2A, B). Thus, the highest BLUPs values for OCFrDW, FrFW, and OLP were obtained in Morocco15, Lebanon15 and Italy16, respectively.

### Influence of temperatures on traits variation

**Figure 4:**
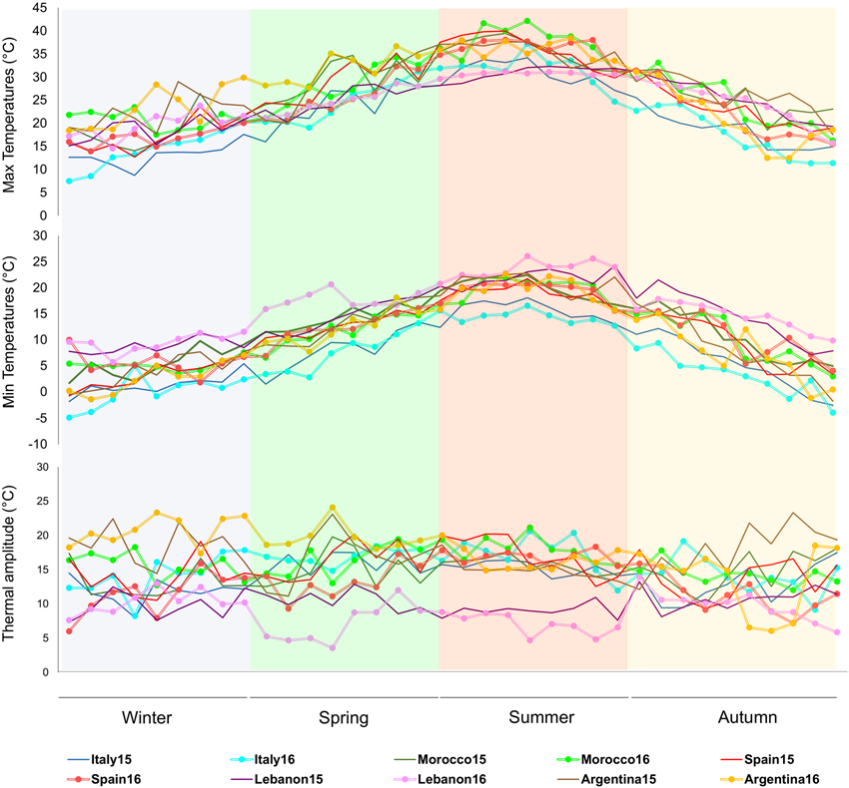
Maximum and minimum average temperature and thermal amplitude (ten days’ interval) for ten environments (five sites x two seasons, each environment showed by different colored lines).

Temperature variation in ten environmental conditions (five collections in two years), showed that the highest average of maximum temperatures (Tmax > 27 °C) was in Morocco15 and Morocco16, (WOGB-INRA, Morocco) and in Argentina15 and Argentina16 (OCC-INTA, Argentina), especially during winter and spring months. Although Italy15 and Italy16 (IOGC, Italy), with about 21 °C on average, had the lowest Tmax, and it was almost the lowest during the year (Fig. 4 and Supplementary Table S5). The highest standard deviation for Tmax during the year was related to Spain15 and Spain16 (WOGB-IFAPA, Spain).

For what concerns the average minimum temperatures, it is interesting to note that in Lebanon15 and Lebanon16 (OC-LARI, Lebanon), minimum temperatures were higher than in other environments, particularly during spring and summer. Furthermore, the Italian environments showed the lowest average of minimum temperatures during the year. Fluctuations between the minimum and maximum daily temperatures reached the highest values in the Argentinean environment, more evident during winter, spring and autumn when the thermal amplitude was higher than 17 °C. The lowest amplitude of temperatures was observed in Lebanon15 and Lebanon16 (10 and 8.6 °C, respectively), during the year and especially in spring and summer (Fig. 4 and Supplementary Table S5).

**Figure 5:**
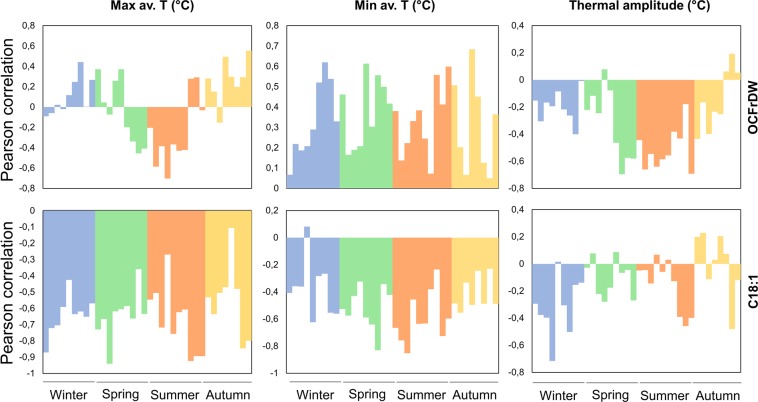
Pearson correlation coefficients of ten-days-average temperatures with OCFrDW (oil content in fruit dry weight) and C18:1(oleic acid) in all accessions and environments.

The whole dataset of 113 cultivars evaluated in nine environments, were used to calculate Pearson’s correlation coefficients between ten-day temperature series and average values of OCFrDW and C18:1 (Fig. 5). Maximum temperature showed a positive correlation with OCFrDW in winter, early spring and autumn, being negatively correlated in late spring and summer. OCFrDW also showed a positive correlation with the minimum temperature throughout the year, being negative with the thermal amplitude. The C18:1 showed a negative correlation with maximum and minimum average temperatures during the year. Negative correlation among C18:1 and temperature amplitude was also obtained in most of the seasons, except in autumn (Fig. 5).

**Figure 6:**
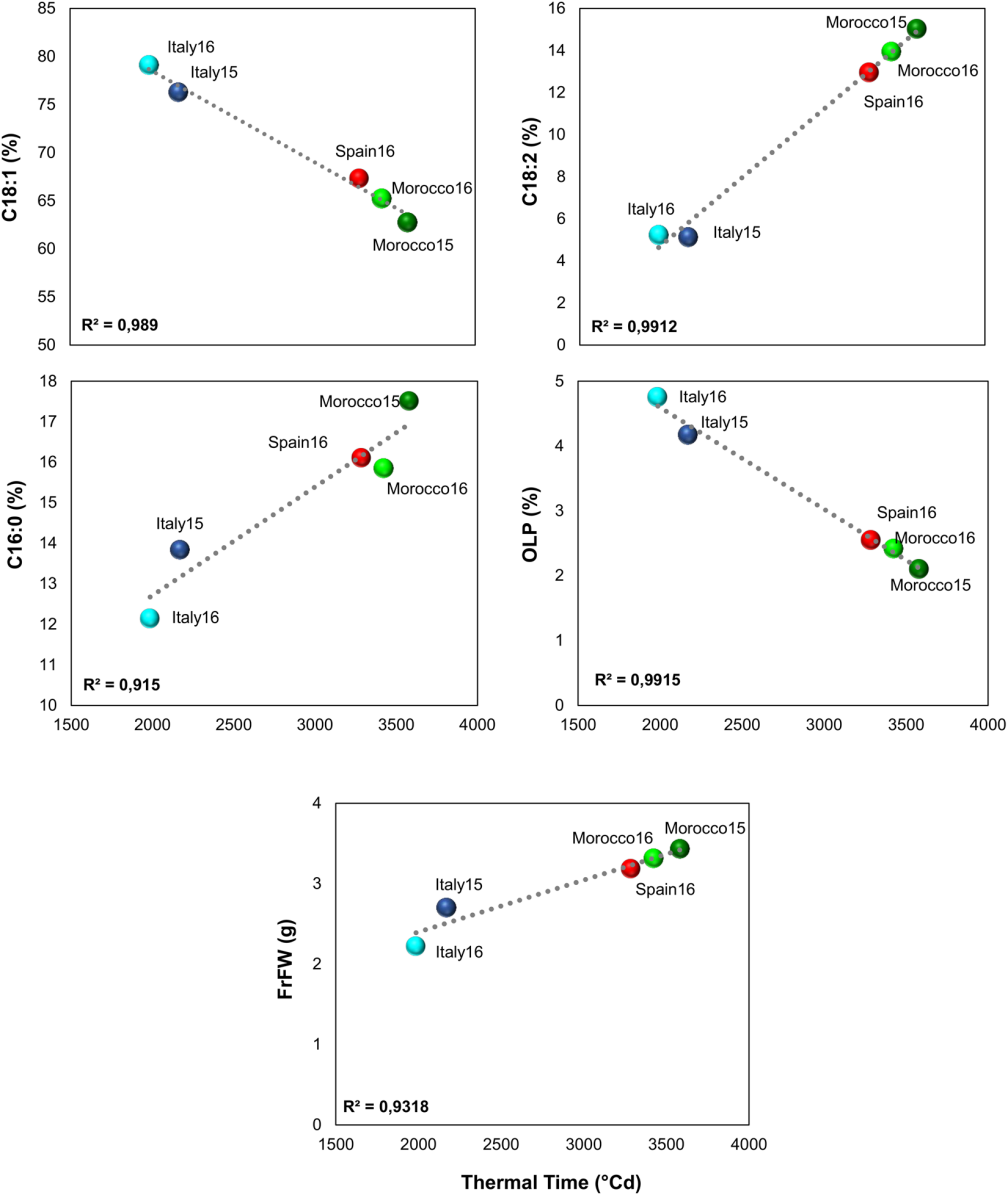
Variation pattern of fatty acids composition and FrFW (fruit fresh weight) as a function of the accumulated thermal time (°Cd) from full flowering to harvesting time.

The accumulation of thermal time for 14 common cultivars in five environments (Fig. 6) showed that the increment of thermal time (TT °C) from 2,000 to 3,500 °C × day was negatively correlated to C18:1 content (R^2^ = 0.99). The highest percentage of this fatty acid was found in Italy15 and Italy16, with 2,000–2,100 TT °C × day and the lowest one was related to Morocco15 with 3,580 TT °C × day. On the contrary, both C18:2 and C16:0 showed positive correlations with TT °C × day (R^2^ = 0.99 and R^2^ = 0.92, respectively). As expected, OLP had the same accumulation pattern as C18:1, with R^2^ = −0.99. A high positive correlation was also found between FrFW and TT °C × day from full flowering time to harvesting time (R^2^ = 0.93). In fact, the lowest FrFW was related to Italy15 and Italy16 and the highest FrFW was achieved in Morocco15. There was no correlation of OCFrDW and FrM with TT °C (Fig. 6).

## Discussion

Trait variation may be due to heritable differences among cultivars or it may be the result of phenotypic plasticity in trait values across varying environmental conditions^58–60^. Understanding which of these sources is responsible for trait variability is crucial for predicting plant response to various temperatures and water regimes^16,61^. The G × E interaction has been largely studied in plant breeding as a mean of producing new cultivars with stable and superior phenotypes^62–65^. G × E of individual traits has been assessed in numerous plants: annual species, such as wheat, rice, Arabidopsis and soybean^66–68^; forest trees, as poplar and pine^68,69^; and, to a lesser extent, fruit trees, including apple, wild cherry, and blueberry^68–73^.

In the present study, the effect of environment and genotype on trait plasticity was studied separately on a set of 14 cultivars simultaneously present in six different environments (three locations per two seasons). The degree of variation was analyzed for seven traits, like oil content and fatty acids composition, as main characters of interest for the agronomical value of cultivars and breeding progenies^18,41,42,74–76^. All analyzed cultivars showed different levels of plasticity for each trait, i.e. different environmental influence was observed for each cultivar and trait combination. ‘Arbequina’, one of the most widely cultivated olive varieties in the world, showed the lowest variation for oil content over the six environmental conditions, highlighting its exceptional capacity for oil yield production in different environments. The two cultivars ‘Picual’ and ‘Koroneiki’, with a vast cultivated area, especially in Spain and Greece, respectively, had their maximum fitness for oleic acid (high and stable values) over all environments. Among all analyzed cultivars, ‘Bianchera’, showed high stability for almost all the evaluated traits, while the opposite was observed for cv. Moraiolo. This may indicate that cv. Bianchera possesses higher adaptive capacity, for the studied traits, expressed as the highest global fitness averaged over the environments^13,77^, rather than the highest fitness in each environment separately^78,79^ respect to cv. Moraiolo.

An important goal of crop improvement should be to minimize unfavorable G × E interaction and allow advantageous dynamic trait responses^9,80^. Considering the importance and the economic impact of the olive oil and table olives production, it is becoming urgent to characterize the wide variability of this species, in order to identify those with a stable response in different environments, especially for important traits like oil content and fruit size. The complex effect of genotype, environment and their interaction on trait plasticity has been analyzed on 113 olive cultivars in five sites for two years, for a total of nine different environments. It represents the first concerted effort to gather information on the phenotype plasticity of a particularly large and representative sample of the entire variability of cultivated olive germplasm. Traits of high agricultural importance, related to fruit and olive oil composition, were taken under consideration.

A high variance in the whole dataset for fruit fresh weight due to the genetic background^81^ and G × E effect for oil content has been observed, while similar high genetic and environmental variance was observed for fatty acids composition. In accordance, G × E interaction was significant for maximum oil content and rate of accumulation, with similar or even higher variance component than the genetic variance, as reported by Navas-Lopez *et al*.^82^. Consequently, high values of broad-sense heritability were found for fruit fresh weight and low values for oil content, as previously reported from the analysis of variability between and within olive breeding progenies^83^. Similarly, the high heritability values obtained for fatty acids and mono-unsaturated fatty acid ratio (OLP) were also reported from studies in olive progenies^84^. Moreover, the correlation between BLUP values extracted from REML models suggested the possibilities for simultaneous selection for oil content and fruit fresh weight, while the opposite was found for oil content and mono-unsaturated fatty acid ratio. These results should be considered in further comparative trials and future breeding programs, to develop molecular tools useful as early selection criteria. Thus, phenotyping of large olive germplasm set under different environments should lead to a specific characterization of loci of interest with respect to classic approaches based on association or QTL mapping.

The correlation analysis between temperature and oil content and composition demonstrated that the highest values of maximum average temperature during the colder months of the year are positively correlated with an increase of oil content, but correlation becomes negative if temperatures rise substantially during the summer. High values of maximum and minimum temperatures affected negatively the percentage of oleic acid^85,86^, particularly if they occurred during the warmer months when fruits are developing and oil is accumulating in the mesocarp. High thermal amplitude during summer decreased the quantity of oil on dry weight and negatively affected the quantity of oleic acid during the colder months^57^. In fact, in the Argentinean environments, the oleic acid content was at the minimum level over all environments. Thus, olive oils arising from warm areas had consistently lower oleic acid content and higher mono-unsaturated fatty acid ratio, independently from cultivars, as previously observed by the analysis of a restricted set of samples and environments^37,55^. On the other hand, in environments with moderate minimum and maximum temperatures, without significant thermal range variability, as in Italy and Lebanon, oleic acid might go to the maximum levels, confirming other evidence obtained in some Italian environments^37,87^. The spread of olive orchards out of the Mediterranean area towards new habitats, completely different from the original ones, is limiting the ability of the species to survive and fructify^3^. Only few cultivars, as is the case of ‘Arbequina’ and some others used in new intensive groves, are able to yield consistently in the new environmental conditions, but often changing negatively their fatty acid profiles^3,53,55^. It has been widely pointed out that summer temperatures differently influence the fatty acids composition of olive oil^37,55,88,89^. It has also been shown that an increase in mean daily temperature, above 25 °C, has a negative effect on fruit dry weight, and a linear decrease both in fruit oil concentration and oleic acid proportion observed over the range of 16–32 °C^1,23^.

From a breeding perspective, it has been suggested that selection criteria should consider adaptive plasticity, particularly in relation to adaptation to climate changes^90^. Therefore, the use of cultivars with high stability, showing interesting traits such as high values of oil yield and oleic acid content, can decrease fluctuations in olive oil production and standard quality. The richness of olive germplasm should be thoroughly protected, conserving cultivars with phenotypic specializations, that would be able to buffer future environmental extremes due to climate and land-use changes^91^.

The present study indicates the need to understand how much phenotypic variability can be mainly attributed to genetic control, in order to enhance the prediction of crop performance across diverse environments.

The study offers useful information on the varieties best suited to each environment, depending on the desired trait. These studies on olive germplasm and comparative trials under different environments are essential to predict the behavior of specific varieties in view of widening the area of olive cultivation and future changes in climate scenarios, particularly regarding new environments not previously experienced by the olive crop.

The results obtained in this work should be taken into account to evaluate both genetic and environmental variance components for the traits mainly related to the product composition. Our study revealed complex patterns of phenotypic plasticity in the most important olive traits. Genotype effect on the plasticity of traits among cultivars, in addition to variation in mean trait values, may thus form a component of the adaptation to different climates in this species. The availability of extensive genetic and traits variation among olive cultivars, the knowledge on the response of fatty acids composition to temperature, and the new information on the genomic composition of the species, will facilitate, in the near future, development of new olive genotypes well adapted to different environments through genomics-assisted breeding.

## Materials and Methods

### Plant material

Fruit samples were collected from trees of cultivars available in five olive germplasm collections located in Italy, Spain, Morocco, Lebanon, and Argentina. Thus, the following collections were included in the study: (i) the World Olive Germplasm Bank (WOGB-IFAPA) of IFAPA (Cordoba, Spain); (ii) the World Olive Germplasm Bank of INRA (WOGB-INRA) (Tassaout, Marrakech, Morocco); (iii) the International Olive Germplasm Collection (IOGC) of Zagaria (Enna, Italy); (iv) the Olive Collection of LARI (OC-LARI) (Abdeh, Lebanon); and (v) the Olive Cultivar Collection of INTA (OCC-INTA) (San Juan, Argentina), to represent non-Mediterranean climate conditions (Supplementary Table S6). The combination of five collection locations and two growing seasons (2015 and 2016) represent nine different environments, (only 2016 data were analyzed from INTA Collection). These environments are represented by: Italy15 and Italy16, related to IOGC in Italy; Morocco15 and Morocco16, related to WOGB-INRA in Morocco; Spain15 and Spain16, correspond to WOGB-IFAPA Spain; Lebanon15 and Lebanon16, belong to OC-LARI Lebanon and Argentina16 related to OCC-INTA, Argentina (Supplementary Table S7). Each collection contributed a different number of cultivars, based on the available genotypes, the economic and ecological importance and the coexistence of the same cultivars in multiple collections. Because of these criteria, the following cultivars were provided by each collection: 57 from WOGB-IFAPA Spain, 65 from WOGB-INRA Morocco, 63 from IOGC Italy, 33 from OC-LARI Lebanon and 12 from OCC-INTA Argentina. The plant material under study included a total of 408 accessions belonging to 113 olive cultivars originating from 16 different countries (Supplementary Table S4). The genetic identity of the selected cultivars was verified through a comparison of each molecular profile based on a revision of all olive collections involved in the European MSCA Before project (data under publication) and by comparison with previously published data from the main olive collections and databases^24,92,93^. A total of 816 trees were sampled, each accession being represented by two trees for each environment. Fourteen cultivars were in common between IOGC, WOGB-INRA and WOGB-IFAPA germplasm collections.

Two kilograms of fruits were harvested from each of the two selected trees. The fruits were randomly chosen around the canopy and promptly transferred to the laboratory. From each sample, 25 g of fruits were weighed before and after fruit drying for the fresh weight (FrFW) and fruit moisture (FrM), all others were conserved at −20 °C. The harvesting time in the Mediterranean countries was set in November, as the usual harvesting time in this hemisphere, while in Argentina fruits were collected in May.

### Measuring oil content and fatty acids profiling

Three sub-samples of around 25 g of fruits were randomly selected to measure FrFW. They were dried in a forced-air oven at 105 °C for 42 h to ensure dehydration and to determine FrM. The same samples have been used to measure the oil content using an NMR Fat Analyzer Bruker Series NMS 100 Minispec (Bruker Optik GmbH, Ettlingen, Germany). The results were expressed as a percentage of dry fruit weight (OCFrDW). The samples were prepared through the same protocol in each collection and all the NMR analysis was performed in one laboratory.

FAs composition was analyzed by FAs methylation of fruit flesh (fruit epicarp and mesocarp)^94^ with minor changes. 50–100 mg of the flesh were placed in a reaction tube. The methylation solution was prepared by methanol, toluene, 2,2-dimethoxypropane and sulfuric acid in a 39:20:5:2 proportion. In each reaction tube, 4 ml of the methylation solution and 2 ml of heptane were added. Tubes were then transferred in a water bath for two hours to 80 °C when solution temperature reached to the room temperature, 1.5 ml of supernatant were transferred to the gas-chromatograph tube. Separation of fatty acid methyl esters was carried out by Gas–Liquid Chromatography (GLC) with a Split injector and flame ionization detector, using similar equipment and conditions in the different laboratories involved in the work. Fatty acids monitored in this study include palmitic acid (C16:0), oleic acid (C18:1) and linoleic acid (C18:2) expressed as a percentage of total FAs and oleic/(linoleic + palmitic) acids ratio (OLP).

### Statistical analysis

Statistical descriptive analyses of the different traits were performed for one balanced and for the whole unbalanced datasets. The balanced dataset includes 14 common cultivars available in the three collections IOGC, WOGB-INRA and WOGB-IFAPA located in Italy, Morocco and Spain, respectively and two seasons (six environments). For these cultivars, variability for seven traits was calculated through Violin Plot Statlet (StatGraphics Centurion, www.statgraphics.com).

The quantitative estimators of phenotypic plasticity^95,96^, were calculated in two simplified approaches as follows; 1) The environmental effect on trait plasticity in a common set of cultivars was calculated through the coefficient of variation for each trait in each environment (CV_trait_); 2) The genotype effect on trait plasticity in a common set of cultivars was calculated through the coefficient of variation over the environments (calculated as standard deviation of means/mean of means, CV_m_).

The complex effect of genotype, environment and their interaction on trait plasticity of all studied cultivars was carried out on the unbalanced dataset, including all 113 genotypes from five collections and two seasons (nine environments). Trait variation in each environment, as well as overall environments, was calculated as previously described.

Variance components were then estimated according to the statistical model:

$${{\rm{P}}}_{{\rm{ijk}}}=\mu +{{\rm{G}}}_{{\rm{i}}}+{{\rm{E}}}_{{\rm{j}}}+{({\rm{G}}\times {\rm{E}})}_{{\rm{ij}}}+{{\rm{\varepsilon }}}_{{\rm{ijk}}}$$

where P_ijk_ was the phenotypic value of the *k* tree of the *i* genotype in the *j* environment; µ the overall mean of the progeny; G_i_ a random effect contributed by the *i* genotype; E_j_ a random effect of the *j* environment; (G × E)_ij_ the interaction between the *i* genotype and the *j* environment; and ε_ijk_ was the random residual error effect.

From this model, variance components among genotypes (σ²_G_), among environments (σ²_E_), associated with the G × E interaction (σ²_GE_), and residual error effect for the measured samples (σ²_ε_), were obtained. Broad-sense heritability (H²) for all evaluated traits was estimated as the ratio between genotypic and phenotypic variances: σ²_G_/(σ²_G_ + σ²_GE_ + σ²_ε_). Best linear unbiased prediction (BLUP) values for genotypes and environments were also estimated from these models. Analyses were performed using R version 3.5.0 software, with REML estimation method under lme4 package for developing mixed linear models (R Core Team, 2018).

### The effect of temperature and thermal time on trait variation

Climate data were registered in each collection site along the years 2015 and 2016 when fruits were collected. Among climate variables, temperature data were selected to test their effect on phenotypic plasticity of the functional traits under analysis. Daily average maximum and average minimum temperatures, as well as thermal amplitude, were calculated from hourly data for each site. In this case, data on a new environment (Argentina15) has been included, being 10 the total number of environments under study.

To calculate the accumulation of thermal time in each environment for the 14 common cultivars, linear or bilinear functions were fitted to the relationships between three main fatty acids (C18:1, C18:2 and C16:0), OCFrDW, FrM, FrFW and OLP with thermal time (TT °C × day, from 7 °C to 40 °C). Thermal time was registered from full flowering time to the harvesting time, using the single sine, horizontal cut-off method^88,97^. Five environments were considered in this case as Spain15 was excluded from the analysis due to missing flowering time data. Pearson correlation coefficient was used to analyze the correlation between climate data (Max, Min, and amplitude temperatures) and average values of the studied traits in each environment.

## Supplementary information

Supplementary data

## Data Availability

All information about protocols, materials, and methods described in this manuscript are sufficient to replicate the research. Moreover, we are available to share or give new information to the editor and reviewers, if necessary.
