# Supplementary material for: Plasticity of fruit and oil traits in olive among different environments
Source: Sci Rep. 2019 Nov 18;9:16968. doi: 10.1038/s41598-019-53169-3 (PMC6861299; doi:10.1038/s41598-019-53169-3)
Supplement: Supplementary file 1 — Supplementary data [file 41598_2019_53169_MOESM1_ESM.pdf]

Supplementary data

Plasticity of fruit and oil traits in olive among different environments

Soraya Mousavi[1,2], Raul de la Rosa[3], Abdelmajid Moukhli[4], Milad El Riachy[5], Roberto Mariotti[2], Mariela Torres[6], Pierluigi Pierantozzi[6], Vitale Stanzione[1], Valerio Mastio[1], Hayat Zaher[4], Abderraouf El Antari[4], Salam Ayoub[7], Faten Dandachi[5], Hiyam Youssef[5], Nikolas Aggelou[8], Cibeles Contreras[6], Damián Maestri[9], Angjelina Belaj[3], Marina Bufacchi[1], Luciana Baldoni[2], Lorenzo Leon[3]

[1]CNR - Institute for Agricultural and Forest Systems of the Mediterranean, 06128 Perugia, Italy. [2]CNR - Institute of Biosciences and Bioresources, 06128 Perugia, Italy. [3]IFAPA - Centro Alameda del Obispo, Córdoba, Spain. [4]INRA - CRRA, Marrakech-Safi, BP 533, Marrakech, Morocco. [5]LARI - Lebanese Agricultural Research Institute Tal Amara, Bekaa, Lebanon. [6]Estación Experimental Agropecuaria San Juan (EEA INTA San Juan), and CONICET. Ing. Marcos Zalazar (Calle 11) y Vidart. Villa Aberastain, Pocito, 5427 San Juan, Argentina. [7]National Agricultural Research Center (NARC), Baqa, 19381 Jordan. [8]MAICh - Department of Horticultural Genetics and Biotechnology, 73100 Chania-Crete, Greece. [9]Instituto Multidisciplinario de Biología Vegetal (IMBIV, CONICET-UNC). Av. Vélez Sarsfield 1611, X5016GCA Córdoba, Argentina

Author for correspondence: Marina Bufacchi

Email: *marina.bufacchi@cnr.it*

Tel: +39 075 5015538

Email addresses:

Soraya Mousavi: soraya.mousavi@ibbr.cnr.it

Raul de la Rosa: raul.rosa@juntadeandalucia.es

Abdelmajid Moukhli: moukhli.abdelmajid@gmail.com

Milad El Riachy: mriachy@lari.gov.lb

Roberto Mariotti: roberto.mariotti@ibbr.cnr.it

Mariela Torres: torres.mariela@inta.gob.ar

Pierluigi Pierantozzi: pierantozzi.pierluigi@inta.gob.ar

Vitale Stanzione: vitale.stanzione@cnr.it

Valerio Mastio: valerio.mastio@hotmail.it

Hayat Zaher: hayatzaher@yahoo.fr

Abderraouf El Antari: aelantari@yahoo.fr

Salam Ayoub: salam.ayoub@narc.gov.jo

Faten Dandachi: fatendandachi@hotmail.com

Hiyam Youssef: hiyamy@hotmail.com

Nikolas Aggelou: aggelou.nikolas@gmail.com

Cibeles Contereras: contreras.cibeles@inta.gob.ar

Damián Maestri: dmaestri@unc.edu.ar

Angjelina Belaj: angjelina.belaj@juntadeandalucia.es

Marina Bufacchi: marina.bufacchi@cnr.it

Luciana Baldoni: luciana.baldoni@ibbr.cnr.it

Lorenzo Leon: lorenzo.leon@juntadeandalucia.es

Supplementary Figure S1 and S2; Supplementary Tables S1-S7

Figure S1 The best linear unbiased prediction (BLUP) graph for the effect of genotype on OCFrDW (oil content in fruit dry weight) *vs* OLP (oleic/(linoleic + palmitic) acids ratio), interactions.

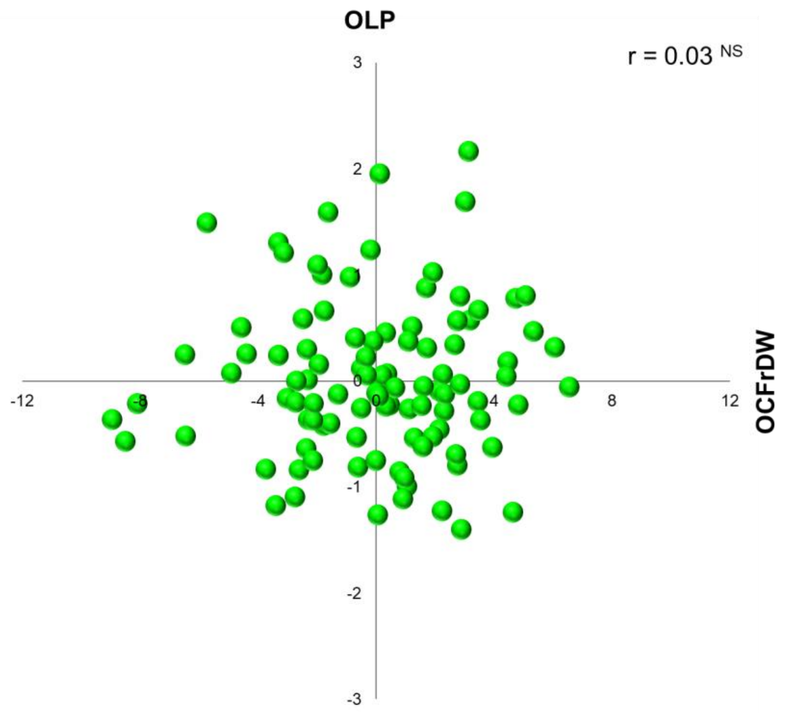

Figure S2 The best linear unbiased prediction (BLUP) graph for the effect of environment on OCFrDW (oil content in fruit dry weight) vs FrFW (fruit fresh weight) and OLP (oleic/(linoleic + palmitic) acids ratio) vs OCFrDW interactions.

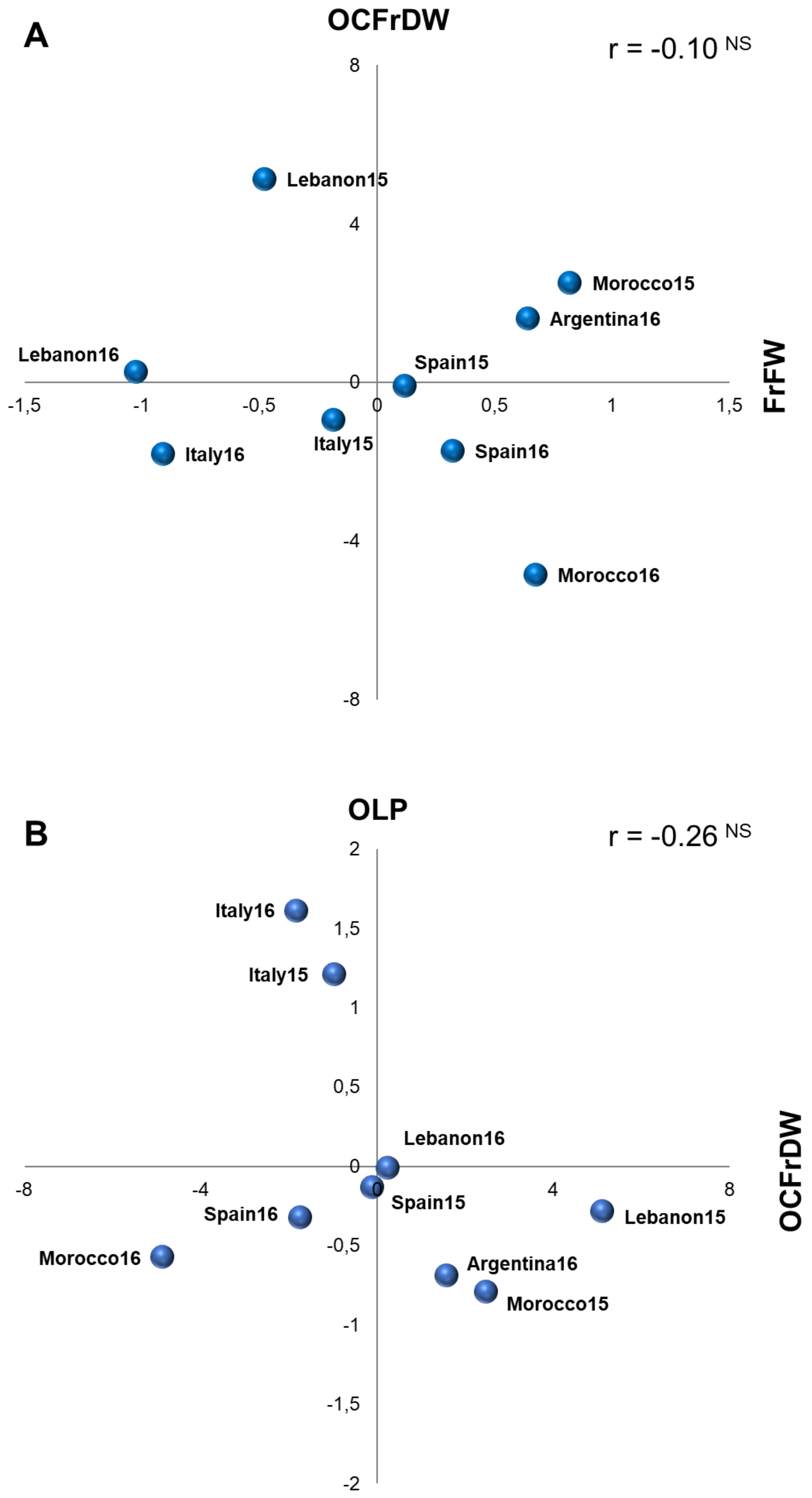

Table S1 Trait variability among 14 olive cultivars in each environment. The trait variation was calculated as CVtrait %.

Trait	Italy15	Italy16	Morocco15	Morocco16	Spain15	Spain16
**FrFW**[†]						
Min	0.6	0.9	1.1	0.9	0.9	0.7
Max	5.6	4.9	8.5	6.2	6.6	5.6
Mean	2.7	2.2	3.4	3.3	3.0	3.2
CV_{trait}[a]	**48.6**	**53.8**	**55.0**	**43.0**	**49.4**	**42.5**
**FrM**						
Min	57.0	40.9	40.5	46.4	55.4	57.9
Max	71.7	57.7	64.3	65.4	72.1	68.7
Mean	62.3	48.7	53.7	57.4	63.5	63.3
CV_{trait}	**6.5**	**9.4**	**12.8**	**10.0**	**7.3**	**4.7**
**OCFrDW**						
Min	29.2	29.5	30.5	25.9	36.8	29.3
Max	49.0	51.2	55.1	45.9	61.4	55.2
Mean	41.0	40.1	45.8	37.3	44.9	41.3
CV_{trait}	**15.8**	**15.1**	**15.0**	**17.8**	**14.6**	**15.4**
**C16:0**						
Min	10.7	10.1	15.0	12.6	12.4	12.8
Max	16.7	14.8	21.4	21.7	21.2	21.6
Mean	13.8	12.1	17.5	15.9	15.7	16.1
CV_{trait}	**13.5**	**11.0**	**10.5**	**15.9**	**14.6**	**14.7**
**C18:1**						
Min	69.0	69.7	51.0	53.9	48.9	45.8
Max	80.9	84.0	75.0	76.8	81.6	79.2
Mean	76.3	79.1	62.8	65.3	68.8	67.3
CV_{trait}	**4.7**	**4.6**	**12.2**	**10.5**	**11.7**	**11.7**
**C18:2**						
Min	2.4	1.9	5.1	4.7	2.1	2.9
Max	10.5	11.7	25.8	21.8	25.2	28.4
Mean	5.1	5.2	15.0	14.0	11.5	13.0
CV_{trait}	**38.6**	**44.3**	**41.7**	**38.1**	**51.2**	**46.7**
**OLP**						
Min	2.6	2.6	1.2	1.3	1.1	0.9
Max	5.5	6.6	3.7	4.4	5.6	4.7
Mean	4.2	4.8	2.1	2.4	2.9	2.5
CV_{trait}	**21.2**	**22.4**	**36.9**	**40.7**	**42.6**	**35.7**

[†]FrFW expressed as g of weight, all other values expressed as percentage.

Abbreviations: FrFW, fruit fresh weight, FrM, fruit moisture, OCFrDW, oil content in fruit dry weight, C16:0, palmitic acid, C18:1, oleic acid, C18:2, linoleic acid and OLP, oleic/(linoleic + palmitic) acids ratio.

Table S2 The phenotypic variation of each cultivar in six different environments (the measurements expressed in percentage, except FrFW, which expressed in g). The trait variation was calculated as CVtrait % and the total trait variation over six different environments (CVm %).

Trait	Arbequina	Bianchera	Carolea	Coratina	Empeltre	Frantoio	Koroneiki	Lastovka	Leccino	Maurino	Moraiolo	Oblica	Picual	Salonenque	CV_m
**FrFW**															
Min	1.1	2.7	2.5	1.5	2.5	1.5	0.6	1.3	1.0	1.3	1.4	4.9	1.7	2.0	
Max	2.5	4.0	6.6	3.9	4.1	2.9	1.1	2.8	3.8	2.8	2.8	8.5	6.0	4.5	
Mean	1.7	3.4	4.9	2.9	3.2	2.4	0.8	2.4	2.5	2.2	2.2	6.2	3.4	3.6	
CV_{trait}	**30.9**	**11.9**	**27.9**	**25.9**	**18.5**	**19.4**	**19.8**	**21.4**	**33.8**	**20.7**	**19.5**	**18.3**	**48.6**	**22.3**	**13.77**
**FrM**															
Min	47.1	49.5	40.5	43.4	55.5	40.9	47.4	51.8	47.8	54.8	41.2	54.8	45.6	47.8	
Max	72.1	61.5	64.3	65.4	67.8	59.0	63.8	65.1	66.3	71.7	63.2	68.6	62.8	68.7	
Mean	56.7	57.4	54.7	55.5	62.3	53.3	58.7	60.7	57.4	63.4	52.9	63.9	57.3	59.7	
CV_{trait}	**16.8**	**7.0**	**15.8**	**11.9**	**6.9**	**12.1**	**9.2**	**7.4**	**10.9**	**10.0**	**17.4**	**6.8**	**10.3**	**12.4**	**10.88**
**OCFrDW**															
Min	31.6	34.3	38.7	33.4	33.4	32.9	29.2	26.0	29.5	36.5	25.9	32.9	30.3	40.0	
Max	42.5	61.4	56.4	46.4	43.1	47.7	42.3	47.3	46.2	52.8	53.3	48.2	52.3	47.5	
Mean	39.0	49.6	47.8	41.0	37.7	42.4	34.2	38.0	40.0	42.3	42.1	43.8	41.2	44.9	
CV_{trait}	**9.3**	**16.9**	**13.0**	**12.8**	**8.8**	**11.0**	**16.8**	**20.6**	**14.6**	**14.5**	**19.4**	**11.5**	**16.9**	**5.3**	**8.41**
**C16:0**															
Min	13.7	13.3	12.9	10.1	11.2	11.6	10.6	11.1	13.1	12.9	12.9	10.9	10.8	14.7	
Max	21.7	17.9	18.2	16.7	17.9	17.9	15.4	15.2	16.7	17.5	20.7	18.0	15.0	21.6	
Mean	17.3	15.4	15.9	12.7	15.3	15.0	13.1	13.4	15.6	15.8	17.1	14.2	12.9	19.0	
CV_{trait}	**16.4**	**9.2**	**13.4**	**15.6**	**17.6**	**13.7**	**14.6**	**9.0**	**7.8**	**10.6**	**14.6**	**15.4**	**10.5**	**14.4**	**12.55**
**C18:1**															
Min	58.8	61.7	62.0	51.8	63.5	60.4	73.6	63.0	60.5	62.1	55.2	52.5	74.8	45.8	
Max	76.6	76.2	78.6	83.0	82.8	81.0	84.0	78.4	78.1	77.8	78.3	79.6	83.8	69.7	
Mean	66.7	70.1	69.8	74.5	70.4	71.1	77.8	69.1	71.9	68.9	66.6	66.4	79.4	56.4	
CV_{trait}	**10.6**	**7.0**	**8.5**	**14.0**	**11.3**	**10.0**	**4.5**	**7.1**	**7.9**	**8.2**	**11.7**	**14.7**	**3.7**	**16.8**	**9.04**
**C18:2**															
Min	5.3	5.4	4.3	3.8	2.8	3.6	2.7	7.1	3.6	5.8	5.6	4.9	1.9	10.5	
Max	17.9	14.3	12.9	20.8	16.1	16.6	7.0	17.3	16.3	17.4	19.9	24.9	5.1	28.4	
Mean	12.3	9.2	9.0	8.0	10.5	10.3	4.9	13.0	8.2	11.4	12.5	15.5	3.2	20.0	
CV_{trait}	**38.8**	**38.2**	**31.6**	**75.4**	**52.2**	**46.2**	**31.9**	**33.1**	**49.4**	**37.9**	**42.0**	**48.3**	**39.4**	**33.3**	**40.75**
**OLP**															
Min	1.5	1.9	2.0	1.2	1.9	1.8	3.3	1.9	1.9	1.8	1.4	1.2	3.7	0.9	
Max	4.0	3.9	4.6	6.0	5.9	5.1	6.3	4.3	4.3	4.2	4.2	4.7	6.6	2.6	
Mean	2.5	3.0	3.0	4.1	3.3	3.1	4.5	2.8	3.2	2.7	2.5	2.7	5.1	1.6	
CV_{trait}	**40.5**	**23.7**	**31.3**	**36.5**	**51.9**	**40.4**	**23.9**	**29.8**	**24.2**	**30.4**	**40.0**	**51.7**	**18.8**	**44.8**	**32.65**

Abbreviations: FrFW, fruit fresh weight, FrM, fruit moisture, OCFrDW, oil content in fruit dry weight, C16:0, palmitic acid, C18:1, oleic acid, C18:2, linoleic acid and OLP, oleic/(linoleic + palmitic) acids ratio.

Table S3 Pearson correlation coefficients among the evaluated traits by cultivar. Significant correlation coefficients are indicated in bold.

Cultivar	Trait	FrFW	FrM	OCFrDW	C16:0	C18:1	C18:2
Arbequina	FrM	0.22					
Carolea	FrM	0.34					
Coratina	FrM	0.85					
Empeltre	FrM	0.11					
Frantoio	FrM	0.73					
Bianchera	FrM	0.67					
Koroneiki	FrM	-0.24					
Lastovka	FrM	-0.02					
Leccino	FrM	0.51					
Maurino	FrM	0.76					
Moraiolo	FrM	0.39					
Oblica	FrM	0.38					
Picual	FrM	0.70					
Salonenque	FrM	0.03					
Arbequina	OCFrDW	-0.86	-0.01				
Carolea	OCFrDW	0.58	0.04				
Coratina	OCFrDW	0.68	0.51				
Empeltre	OCFrDW	-0.41	-0.33				
Frantoio	OCFrDW	0.84	0.70				
Bianchera	OCFrDW	0.26	0.17				
Koroneiki	OCFrDW	0.36	-0.50				
Lastovka	OCFrDW	0.66	-0.29				
Leccino	OCFrDW	0.71	0.30				
Maurino	OCFrDW	0.48	-0.03				
Moraiolo	OCFrDW	-0.25	0.04				
Oblica	OCFrDW	-0.90	-0.02				
Picual	OCFrDW	0.92	0.46				
Salonenque	OCFrDW	0.69	0.23				
Arbequina	C16:0	0.86	-0.13	-0.70			
Carolea	C16:0	0.81	-0.18	0.46			
Coratina	C16:0	0.56	0.14	0.62			
Empeltre	C16:0	**-0.96**	-0.04	0.41			
Frantoio	C16:0	0.65	0.39	0.61			
Bianchera	C16:0	0.49	0.28	-0.28			
Koroneiki	C16:0	0.38	0.57	-0.42			
Lastovka	C16:0	-0.29	0.70	-0.15			
Leccino	C16:0	0.75	0.63	0.82			
Maurino	C16:0	0.88	0.48	0.65			
Moraiolo	C16:0	0.51	0.12	0.48			
Oblica	C16:0	**0.92**	0.62	-0.79			
Picual	C16:0	0.90	0.85	0.79			
Salonenque	C16:0	0.59	0.61	0.41			
Arbequina	C18:1	-0.85	-0.06	0.58	**-0.97**		
Carolea	C18:1	-0.84	0.17	-0.43	**-0.99**		
Coratina	C18:1	-0.40	0.09	-0.48	**-0.96**		
Empeltre	C18:1	**0.93**	-0.04	-0.38	**-0.96**		

Frantoio	C18:1	-0.43	-0.33	-0.42	**-0.95**		
Bianchera	C18:1	-0.40	-0.12	0.61	-0.91		
Koroneiki	C18:1	-0.05	-0.74	0.77	-0.87		
Lastovka	C18:1	0.01	-0.81	0.26	-0.88		
Leccino	C18:1	-0.79	0.10	-0.66	-0.49		
Maurino	C18:1	-0.58	-0.20	-0.33	-0.82		
Moraiolo	C18:1	-0.59	0.18	-0.21	-0.90		
Oblica	C18:1	-0.84	-0.61	0.72	**-0.92**		
Picual	C18:1	-0.90	-0.82	-0.68	-0.90		
Salonenque	C18:1	-0.67	-0.66	-0.53	**-0.98**		
Arbequina	C18:2	0.87	0.08	-0.62	**0.96**	**-1.00**	
Carolea	C18:2	0.91	-0.03	0.47	**0.96**	**-0.98**	
Coratina	C18:2	0.37	-0.14	0.54	**0.93**	**-0.98**	
Empeltre	C18:2	-0.89	0.00	0.34	**0.92**	**-0.99**	
Frantoio	C18:2	0.31	0.32	0.37	0.89	**-0.98**	
Bianchera	C18:2	0.39	-0.05	-0.64	0.76	**-0.94**	
Koroneiki	C18:2	0.09	0.58	-0.73	0.86	**-0.96**	
Lastovka	C18:2	0.39	0.75	-0.11	0.61	-0.90	
Leccino	C18:2	0.69	-0.26	0.52	0.30	**-0.97**	
Maurino	C18:2	0.39	0.10	0.08	0.62	**-0.96**	
Moraiolo	C18:2	0.56	-0.25	0.18	0.85	**-0.99**	
Oblica	C18:2	0.77	0.60	-0.67	0.88	**-0.99**	
Picual	C18:2	0.90	0.68	0.73	0.79	**-0.95**	
Salonenque	C18:2	0.71	0.67	0.59	**0.95**	**-0.99**	
Arbequina	OLP	-0.85	-0.10	0.56	**-0.94**	**1.00**	**-1.00**
Carolea	OLP	-0.90	0.03	-0.54	**-0.98**	**0.98**	**-0.99**
Coratina	OLP	-0.51	-0.10	-0.62	**-0.98**	**0.95**	**-0.94**
Empeltre	OLP	**0.93**	0.00	-0.43	**-0.97**	**1.00**	**-0.98**
Frantoio	OLP	-0.45	-0.43	-0.55	**-0.93**	**0.97**	**-0.98**
Bianchera	OLP	-0.52	-0.17	0.54	-0.88	**0.98**	**-0.96**
Koroneiki	OLP	-0.24	-0.69	0.61	**-0.95**	**0.97**	**-0.95**
Lastovka	OLP	-0.11	-0.87	0.25	-0.80	**0.98**	**-0.95**
Leccino	OLP	-0.85	-0.03	-0.74	-0.61	**0.96**	**-0.93**
Maurino	OLP	-0.64	-0.29	-0.31	-0.81	**0.99**	**-0.94**
Moraiolo	OLP	-0.72	0.00	-0.14	-0.91	**0.98**	**-0.96**
Oblica	OLP	-0.71	-0.70	0.57	-0.87	**0.97**	**-0.99**
Picual	OLP	-0.91	-0.89	-0.73	**-0.95**	**0.98**	**-0.92**
Salonenque	OLP	-0.66	-0.64	-0.54	**-0.98**	**1.00**	**-0.98**

Abbreviations: FrFW, fruit fresh weight, FrM, fruit moisture, OCFrDW, oil content in fruit dry weight, C16:0, palmitic acid, C18:1, oleic acid, C18:2, linoleic acid and OLP, oleic/(linoleic + palmitic) acids ratio.

Table S4 List of cultivars collected from nine different environments.

Cultivar	Country of origin	Italy15	Italy16	Morocco15	Morocco16	Spain15	Spain16	Lebanon15	Lebanon16	Argentina16
Adramitini-DA12I	Greece	X	X							
Agrestigna	Italy	X								
Amygdalolia Nana	Greece					X	X			
Arauco	Argentina		X			X	X			X
<u>Arbequina</u>†	Spain	X	X	X	X	X	X			X
Ascolana Tenera	Italy			X	X	X	X	X	X	X
Ayrouni	Lebanon			X						
Ayvalik	Turkey					X	X			
Baladi Ain Baal	Lebanon					X	X			
Baladi Fekhe	Lebanon			X	X					
Baladi Kana	Lebanon								X	
Bella di Cerignola	Italy			X				X		
Bella di Spagna	Italy			X						
<u>Bianchera</u>	Italy	X	X	X	X	X	X	X	X	
Biancolilla	Italy		X	X	X			X	X	
Biancolilla nana	Italy	X	X							
Blanqueta	Spain			X	X	X	X			X
Bosana	Italy					X	X			
Bouteillan	France			X	X	X	X			
Branquita	Portugal	X	X							
Bujuk Topakislak	Turkey	X	X							
Canino	Italy	X	X			X	X	X	X	X
<u>Carolea</u>	Italy	X	X	X	X	X	X			
Chalkidikis	Greece	X	X	X	X					
Changlot Real	Spain	X	X			X				X
Chemlali	Tunisia	X	X							
Chetoui	Tunisia		X	X	X	X	X			
Cipressino	Italy	X	X			X	X			
Cobrancosa	Portugal	X	X	X	X	X				
<u>Coratina</u>	Italy	X	X	X	X	X	X		X	
Cornicabra	Spain			X	X	X				
Domat	Turkey	X	X				X			
Dritta	Italy	X	X	X	X					
<u>Empeltre</u>	Spain	X	X	X	X	X	X			X
Fishomi	Iran	X	X			X	X			
<u>Frantoio</u>	Italy	X	X	X	X	X	X	X	X	X
Galega Vulgar	Portugal			X	X	X	X			
Galego	Portugal		X							

Gordal Sevillana	Spain	X	X	X	X	X	X			
Grappolo	Italy			X						
Grossa di spagna	Italy			X						
Hojiblanca	Spain	X	X	X	X	X		X	X	
Itrana	Italy			X	X	X	X			
Izmir Sofralik	Turkey		X				X			
Kalamata	Greece	X	X					X	X	
Kalokerida	Greece			X	X	X	X			
Kerkiras	Greece					X	X			
Konservolia	Greece	X	X			X	X	X	X	
Koroneiki	Greece	X	X	X	X	X	X			
Lastovka	Croatia	X	X	X	X	X	X			
Leccino	Italy	X	X	X	X	X	X	X	X	X
Leccio del Corno	Italy		X	X	X			X	X	X
Lechin de Sevilla	Spain	X	X	X		X	X	X	X	
Lucques	France					X	X			
Madural	Portugal			X	X					
Majorca	Italy		X	X	X					
Manzanilla Cacerena	Spain	X		X	X	X	X			
Manzanilla de Agua	Spain			X						
Manzanilla de Jaen	Spain	X								
Manzanilla de Sevilla	Spain	X	X	X	X	X	X		X	
Massabi	Syria							X	X	
Mastoidis	Greece			X	X					
Maurino	Italy	X	X	X	X	X	X	X	X	X
Megaritiki	Greece	X								
Memecik	Turkey					X				
Meski	Tunisia	X	X	X		X	X			
Moraiolo	Italy	X	X	X	X	X	X	X	X	
Moresca	Italy	X	X	X	X					
Morisca	Spain		X	X	X	X				
Morrut	Spain	X	X							
Nabali	Jordan							X	X	
Nocellara del Belice	Italy			X	X			X	X	
Nociara	Italy	X	X	X	X			X	X	
Oblica	Croatia	X	X	X	X	X	X			
Ottobratica	Italy			X	X					
Ottobratica-DA12I	Italy	X	X							
Passalunara	Italy	X	X							
Pendolino	Italy	X	X	X	X	X	X	X	X	
Peranzana	Italy	X	X							

Picholine du Gard	France			X	X					
Picholine Languedoc	France	X	X	X	X	X	X	X	X	
Picholine Marocaine	Morocco	X	X	X	X	X		X	X	
Picual	Spain	X	X	X	X	X	X	X	X	
Picudo	Spain	X	X	X	X	X	X			
Rachati	Greece					X	X			
Ravece	Italy	X								
Remnani	Lebanon			X				X	X	
Rkhami	Tunisia			X	X					
Rotondella	Italy			X	X					
Roumani Kana	Lebanon			X	X					
Rowghani	Iran					X				
Rowghani-I	Iran	X								
Salonenque	France	X	X	X	X	X	X			
Santa Caterina	Italy								X	
Sant Agostino	Italy			X	X			X	X	
Sevillenca	Spain			X	X	X	X			
Shami	Jordan			X	X					
Sigoise	Algeria			X	X			X	X	
Sorani	Syria							X	X	
Souri	Lebanon			X						
Tanche	France	X	X							
Toffahi	Egypt			X	X	X		X		
Tonda Iblea	Italy	X	X						X	
Uovo di Piccione	Italy		X	X	X					
Uslu	Turkey					X	X			
Valanolia	Greece					X	X			
Vera	Spain			X	X	X	X			
Vera-Umbria	Italy		X							
Verdale	France					X	X	X	X	
Verdial de velez Malaga	Spain	X	X							
Villalonga	Spain									X
Zaituna	Italy	X								
Zaity	Syria	X	X							
**Total accessions/environment**		**55**	**58**	**65**	**56**	**55**	**48**	**28**	**31**	**12**
**Total cultivars/collection**	**16**	**63**		**65**		**57**		**33**		**12**

[†] Underlined cultivars represent those sampled from three collections (Italy, Morocco and Spain) and from two years (six environmental conditions).

Table S5 The temperature variation in ten different environments.

Tempereature (°C)	Italy15	Italy16	Morocco15	Morocco16	Spain15	Spain16	Lebanon15	Lebanon16	Argentina15[†]	Argentina16
**Maximum**										
Average annual Tmax	21.4	21.8	27.1	27.6	26.2	25.2	24.9	24.8	28.4	27.8
±SD	7.3	7.9	7.2	7.5	7.9	8.1	5.2	4.9	6.3	7.5
**Minimum**										
Average annual Tmin	7.5	6.2	12.4	11.6	11.0	12.0	14.9	16.2	11.3	10.3
±SD	6.2	6.2	6.1	6.0	6.4	5.7	5.5	5.5	7.4	7.2
**Amplitude**										
Average annual Tamp	13.9	15.6	14.6	16.0	15.2	13.2	10.0	8.6	17.1	17.5
±SD	2.5	2.8	2.4	2.2	3.1	3.2	1.6	2.5	3.2	4.1

[†]Data on a new environment (Env10-A15) has been included, being ten the total number of environments under study.

Table S6 Geo-climate data of olive germplasm collections evaluated in the study.

**Germplasm collections**	**Geographical area**	**Rainfall (mm)**		**Altitude (m)**	**Latitude**	**Longitude**	**Irrigated/Rainfed (mm/year)†**
		2015	2016				
IOGC	Enna (Italy)	988	736	767	37°. 30'. 51"	14°. 17'. 46"	rainfed
WOGB-INRA	Tassaout (Morocco)	207	188	465	32°. 01'. 48"	07°. 14'. 24"	224
WOGB-IFAPA	Cordoba (Spain)	337	615	102	37°. 51'. 38"	04°. 48'. 29"	290
OC-LARI	Akkar (Lebanon)	591	279	18	34°. 31'. 14"	35°. 59'. 19"	rainfed
OCC-INTA	San Juan (Argentina)	87	98	591	31°. 31'. 28"	68°. 23'. 52"	600

† The same amount of irrigation was applied for both years of experiment.

Table S7 Specific information of olive germplasm collections evaluated in the study.

Germplasm collection	Trees distance (m)	Tree age	Fertilization regime	Soil type	Disease and pest control
**WOGB-IFAPA**	7x7	32	Yearly fertilization by NPK following leaf analysis	Sandy loam	No significant pest problems are recorded in WOGB-IFAPA collection where the incidence of olive fly is irrelevant. Phytosanitary treatments were applied on the basis of the monitoring of pests and diseases and on the weather conditions.
**WOGB-INRA**	7x4	16	Yearly fertilization by NPK	Silty sandy	In this collection, no significant pest problems are recorded. As a preventive action, insecticides are applied 2 times a year to control the *Prays oleae*, *Euphyllura olivina* and *Bractocera oleae*
**IOGC**	5x6	15	Biannual fertilization by NPK following leaf analysis	Clay sandy	Insecticides are applied 2 times a year if the presence of *Bractocera oleae,* has been confirmed. Treatments with copper-based products in spring and autumn to control *Spilocaea oleaginea*, based on the presence of this disease and weather condition.
**OC-LARI**	5x6	35	Biannual fertilization by NPK, MgSO4 and Iron	Clay sandy	In few cases, treatment for *Bractocera oleae*, *Prays olea* and *Zeuzera pyrina*
**OCC-INTA**	10x10	75	Biannual fertilization applying 500 g N per tree	Sandy loam	In this collection, no significant disease problems are recorded. Particularly, the olive fly does not constitute a pest of interest in this region. As a preventive action, a systemic insecticide is applied twice a year to control *Pollinia pollini*
